# Anxiety, depression and stress among employees of a public higher education institution in São Paulo, Brazil

**DOI:** 10.5327/Z1679443520190472

**Published:** 2019-12-01

**Authors:** Isabela Maia da Cruz Fernandes, Amanda Mendes Ribeiro, Rayana Loch Gomes, Jaqueline Santos Silva Lopes, Luiz Carlos Marques Vanderlei, Roselene Modolo Regueiro Lorençoni

**Affiliations:** 1 Department of Physical Therapy, Universidade Estadual Paulista “Júlio de Mesquita Filho” - Presidente Prudente (SP), Brazil. Universidade Estadual Paulista Júlio de Mesquita Filho Department of Physical Therapy Universidade Estadual Paulista “Júlio de Mesquita Filho” Brazil; 2 Health Sciences, Universidade Federal de Mato Grosso - Cuiabá (MT), Brazil. Universidade Federal de Mato Grosso Health Sciences Universidade Federal de Mato Grosso Brazil; 3 Department of Physical Therapy, Centro Universitário do Vale do Araguaia - Barra do Garças (MT), Brazil. Department of Physical Therapy Centro Universitário do Vale do Araguaia Brazil

**Keywords:** occupational stress, burnout, professional, occupational health, life style, motor activity

## Abstract

**Background:**

Occupational health is increasing in visibility within the scientific community and has become a field of international research and discussions in which occupational stress is described as a possible stressor.

**Objective:**

To analyze the relationship between anxiety and depression symptoms and socioeconomic level among technical-administrative employees of a public university in the state of São Paulo, Brazil.

**Methods:**

The sample comprised 89 participants. Data were obtained through Lipp’s Inventory of Stress Symptoms for adults to identify levels of stress and the Hospital Anxiety and Depression scale. An additional questionnaire was administered to gather information on educational and socioeconomic levels.

**Results:**

About 45% of the participants exhibited symptoms of anxiety and 39% of depression, however, without direct relationship with their socioeconomic level. Among the participants with depression 50% were professors, and among those with depression 38.4% were administrative employees. Stress was more frequent among the participants who had attended higher education (29.6%) and graduate studies (33.3%).

**Conclusion:**

The study results indicate a high prevalence of anxiety and depression regardless of the socioeconomic level of the participants. Stress was more frequent among the participants with higher educational level.

## RESUMO |

**Introdução:**

A saúde do trabalhador ganha destaque em âmbito científico, sendo tema de pesquisas e debates internacionais, apresentando o estresse laboral como potencial agente estressor.

**Objetivo:**

Caracterizar a incidência de sintomatologia referente à ansiedade e à depressão correlacionada aos níveis socioeconômicos em servidores técnico-administrativos de uma universidade estadual do estado de São Paulo.

**Métodos:**

A amostra foi composta de 89 participantes. Os dados foram obtidos por meio do inventário de sintomas de estresse para adultos de Lipp, para detectar o nível de estresse; da escala hospitalar de ansiedade e depressão, para análise de ansiedade e depressão; e de um questionário informativo para detectar o nível educacional e sociodemográfico.

**Resultados:**

Verificou-se que 47% dos participantes apresentaram sintomas de ansiedade e 39% de depressão, não havendo relação direta com o nível socioeconômico. Além disso, observou-se que, entre os indivíduos que apresentavam ansiedade, 50% eram docentes, e, do total de indivíduos com depressão, 38,4% eram do setor administrativo. O estresse encontra-se em maior escala nas categorias ensino superior (29,6%) e pós-graduados (33,3%).

**Conclusões:**

Os achados demonstraram elevada prevalência de ansiedade e depressão em todas as classes socioeconômicas investigadas. Ademais, indivíduos de nível educacional elevado são mais estressados que os de nível educacional inferior.

## INTRODUCTION

Work is a source of satisfaction, as well as the means to meet many physical and psychosocial needs, including self-accomplishment, personal satisfaction, interpersonal relationships and financial return^1^. At the same time, however, work can also be a source of physical and emotional overload with direct impact on health^1^^,^^2^^,^^3^. Within this context, Dejours, a respected researcher in mental health, asserts that work is never neutral, but a cause of health or disease^4^^,^^5^^,^^6^.

According to the World Health Organization, occupational stress has direct impact on health and is associated with signs and symptoms of anxiety, depression and poor performance, as well as with unemployment, workplace violence and absenteeism^7^. Despite the lack of a clear consensus on its comprehension, stress seems to be a physiological response to stressors^6^. Occupational stressors are related to factors such as productivity increase pressure, retaliation, unsafe working conditions, awkward body posture, unavailability of physical training and orientation and abuse from supervisors^8^.

Stress may give rise to a state of continuous tension liable to disrupt homeostasis that causes imbalance in the human body, with various mental and emotional consequences such mental tiredness, difficult concentration, short-term memory loss, anxiety and mood disorders^9^.

As a function of the aforementioned considerations, investigating occupational stress in definite populations of workers is relevant to identify occupational situations to be taken into account in prevention protocols and strategies to reduce the rates of psychosocial impairments. Better quality of life seems to be directly related to better health, job performance and success indicators, as well as to satisfaction and survival^10^^,^^11^^,^^12^. Therefore, the aim of the present study was to investigate the association between anxiety and depression symptoms and socioeconomic level among technical-administrative employees of a public university in the state of São Paulo, Brazil.

## METHODS

### PARTICIPANTS

The sample comprised 89 employees of School of Science and Technology, Universidade Estadual Paulista “Júlio de Mesquita Filho” (SST-UNESP), President Prudente campus, from both sexes (40 men and 49 women) and average age 42.35±10.33 years old.

To facilitate correlation analysis and the discussion of results, the participants were clustered according to their job position categorized as teaching, administration or general services. We considered all the employees currently working and not on vacation during the study period. We did not include employees with difficulty to understand the administered questionnaires, those who refused to respond them and the ones who could not be located at the workplace after three attempts. The exclusion criteria also considered questionnaire forms with missing data, but no occurrence was registered.

### ETHICAL ISSUES

The participants were duly informed about the study aims and procedures and signed an informed consent form. The study was approved by SST-UNESP research ethics committee, ruling no. 103/2011.

### STUDY DESIGN

In the present cross-sectional observational study data collection was performed in the workplace through standardized and previously validated questionnaires. To avoid interferences derived from the circadian rhythm, all the data were gathered at one single time by one single previously trained examiner.

### PROCEDURES

Collected data included age, sex, body weight and height; the latter two were used to calculate the body mass index (BMI) as weight (kg) divided by height (meters) squared.

We obtained the psychological variables of interest from Lipp’s Inventory of Stress Symptoms for adults (ISSL), which was previously validated for assessment of stress symptoms, and administered the Hospital Anxiety and Depression (HAD) scale to investigate anxiety and depression symptoms. We also collected data on the participants’ socioeconomic and educational levels.

ISSL comprises three sections which represent the stages of stress^8^. Administration takes about 10 minutes and might be individual or in groups of up to 20 participants. In case of illiteracy, items may be read aloud by the examiner. Section 1 (alarm) comprises 12 items relative to physical and 3 items to psychological symptoms having occurred in the past 24 hours. Section 2 (resistance and almost exhaustion) comprises 10 items on physical and 5 items on psychological symptoms in the past week. Finally section 3 (exhaustion) comprises 12 items on physical and 11 items on psychological symptoms in the past month^5^. Therefore, ISSL lists 37 physical and 19 psychological symptoms, often repeated and varying only in strength and severity. Exhaustion is defined based on the frequency of section 2 items^5^.

The participants were instructed to select symptoms they had experienced listed in all three sections. Participants who selected seven or more items in section 1 were categorized as in stage of alarm, those who selected 4 or more items in section 2 as in stage of resistance, and those who selected 9 or more items in section 3 as in stage of exhaustion. This interpretation was based on a previous study performed with the same instrument^5^.

### HAD

We administered a version of HAD translated and validated for use in Brazil to investigate anxiety and depression symptoms^11^. This scale comprises 14 multiple choice items distributed across two subscales, one for state anxiety (7 items) and the other for state depression (7 items). The global score ranges from 0 to 21, individuals with score <7 are categorized as without significant anxiety and/or depression symptoms, 8-10 as with mild symptoms, 11-14 moderate symptoms and 15-21 severe symptoms.

### ASSESSMENT OF SOCIOECONOMIC LEVEL

We established the participants’ socioeconomic level according to the criteria formulated by the Brazilian Association of Research and Market Institutes (ABIPEME) categorized as A1, A2, B1, B2, C1, C2, D and E, where A1 is the top category in terms of housing and consumption patterns^12^.

### EDUCATIONAL LEVEL

We administered a questionnaire with eight response options: illiterate, incomplete elementary school, complete elementary school, incomplete secondary school, complete secondary school, incomplete higher education, complete higher education, incomplete graduate studies and complete graduate studies.

### STATISTICAL ANALYSIS

We performed statistical analysis with Statistical Package for the Social Sciences (SPSS) version 1.3. Parametric data were expressed as mean and standard deviation, non-parametric data as median and interquartile range. Correlations with outcomes stress and educational level were investigated by means of the χ^2^ test; the significance level was set to p<0.05.

## RESULTS

Table 1 describes the sample characterization and anthropometric profile. Males predominated only in the group of administrative employees. Workers in the general services department were the oldest (50.6±8.5 years). The study population was rated obese, especially the group of professors.

**Table 1.** Sample anthropometric characterization, Presidente Prudente, São Paulo, Brazil, 2017 (n=89).

	Mean±standard deviation
Age (years)	42.35±10.33
Height (m)	1.66±0.09
Body weight (kg)	75.31±17.3
BMI (kg/m^2^)	26.0581 (6.62)
WC/HC ratio	0.87±0.11
WC (cm)	89.89±15.92
HC (cm)	100.0 (13)

BMI: body mass index; WC: waist circumference; HC: hip circumference.

Table 2 shows the correlation between stress and educational level. The results indicate that the higher the educational level, also higher the level of stress.

**Table 2.** Stress according to educational level, Presidente Prudente, São Paulo, Brazil 2017 (n=89).

Educational level		N	**Rate (%)**
Illiterate	Stress	0	0%
	No stress	0	0%
	Total	0	0%
Incomplete elementary school	Stress	1	25%
	No stress	3	75%
	Total	4	100%
Complete elementary school	Stress	4	50%
	No stress	4	50%
	Total	8	100%
Incomplete secondary school	Stress	0	0%
	No stress	0	0%
	Total	0	0%
Complete secondary school	Stress	4	21.1%
	No stress	15	78.9%
	Total	19	100%
Incomplete higher education	Stress	0	0%
	No stress	0	0%
	Total	0	0%
Complete higher education	Stress	7	22.6%
	No stress	24	77.4%
	Total	31	100%
Incomplete graduate studies	Stress	0	0%
	No stress	0	0%
	Total	0	0%
Complete graduate studies	Stress	8	29.6%
	No stress	19	70.4%
	Total	27	100%

Table 3 evidences the relationship between stress symptoms and educational levels; no statistically significant association was found in this regard.

**Table 3.** Correlation between stress and educational level, Presidente Prudente, São Paulo, Brazil, 2017 (n=89).

Stress	Educational level					p
	Incomplete elementary	Complete elementary	Secondary	Higher education	Graduate studies	
No	4.6%	6.2%	23.1%	36.9%	29.2%	0.731
Yes	4.2%	16.7%	16.7%	29.6%	33.3%	

Table 4 describes the rates of anxiety and depression symptoms according to socioeconomic level and Table 5 according to job position. The results indicate that the rate of anxiety was highest for class B1 and lowest for A2. In turn, depression was most frequent among the group of professors.

**Table 4.** Anxiety and depression symptoms according to socioeconomic level, Presidente Prudente, São Paulo, Brazil 2017 (n=89).

Socioeconomic class	Anxiety			Depression		
	Unlikely	Possible	Probable	Unlikely	Possible	Probable
A1	2.3%	-	-	-	-	2.3%
A2	23.5%	11.7%	4.6%	26%	9.3%	4.6%
B1	9.3%	13.9%	2.3%	11.7%	6.9%	6.9%
B2	11.7%	2.3%	4.6%	13.9%	4.6%	-
C1	2.3%	2.3%	2.3%	-	2.3%	4.6%
C2	4.6%	-	-	4.6%	-	-
D	2.3%	-	-	2.3%	-	-
E	-	-	-	-	-	-

**Table 5.** Anxiety and depression rates according to job position, Presidente Prudente, São Paulo, Brazil 2017 (n=89).

	Anxiety			Depression		
	Unlikely	Possible	Probable	Unlikely	Possible	Probable
Administration	61,6%	30,8%	7,6%	61,6%	23,1%	15,3%
Teaching	50%	31,2%	18,8%	62,5%	25%	12,5%
General services	53,3%	20%	26,7%	60%	20%	20%

## DISCUSSION

As main results of the present study, we found a high prevalence of anxiety and depression symptoms among the analyzed population. Adding together the participants categorized as with possible and probable symptoms, the rates were 47% for anxiety and 39% for depression. However, our data do not evidence a direct relationship between anxiety or depression symptoms and socioeconomic level or job position. These findings therefore indicate that stress does not depend on any of these two variables. Nevertheless, professors met most often the criteria for anxiety and the administrative employees those for depression.

The frequency of anxiety and depression symptoms is increasing in modern society and early diagnosis might help prevent episodes of emotional crisis^13^^,^^14^^,^^15^. One study that investigated stress-related signs and symptoms among adolescents found that proper identification and modification contributed to minimize future exacerbations^16^. In turn, after analyzing 326 participants clustered into three groups - young, middle-aged and older adults - Wilkowska-Chmielewska et al.^17^ concluded that early diagnosis is determinant to avoid the progression of depression symptoms and episodes.

Within the current cultural context, it is assumed that workers with jobs that involve high physical demands and lower salary, as e.g. in general services, exhibit higher rates of anxiety and depression. However, this was not the case in the present study, since the rates of such symptoms were similar in all three groups. Adding together the participants categorized as with possible or probable symptoms, the highest rate of anxiety corresponded to the group of professors (50%) and that of depression to the administrative employees (38.4%). The emotional overload to which teachers are exposed as a function of continuous stressors in the workplace is well documented in the literature.

Anxiety and depression might be related to job position and increase or decrease as a function of job tasks. Several studies indicate that the most common complaints among teachers are related to excessive use of voice, body posture, psychosomatic or mental health problems. Among administrative employees, pressure and responsibility are some of the most influential factors. In turn, workers in jobs with high physical demands are exposed to higher risk of disease and death when demands become disproportionate, thus increasing the risk of lesions, a situation that is considered a cause of considerable concern^18^.

In the present study, the participants with the highest levels of stress also exhibited the highest rates of anxiety and depression. Beyond job position, these findings may be related to many other factors, such as the personal lives of workers. Thus being, additional studies are needed to identify such factors for the purpose of prevention and therapeutic recommendations.

The degree of job satisfaction may vary considerably among teachers and also among different specialties within one and the same occupational group. Similarly, the level of occupational stress is influenced by the tasks workers perform^19^^,^^20^^,^^21^^,^^22^. Based on the experience narrative method, Fernandes et al.^23^ analyzed the impact of occupational stress on the lives of nurses. In our study, the participants’ working conditions - including work environment, productivity-to-salary ratio, working and rest hours and dietary intake - were the aspects rated as most stressful.

A recent study analyzed stress among employees of a higher education institution. The results showed that almost half of the participants had depression and that there was not any relationship between occurrence of mental disorders and age (37.83±12.05 years old, on average). In our study, the rate of stress was lower, perhaps due to the job stability of civil servants, which ensures some degree of comfort and ease in regard to the future.

Graduate studies or specialty accreditation used to be differential factors in the labor market, but in the present time they are increasingly becoming mandatory for some job positions^21^^,^^22^. Within this context, one may assume that well-prepared workers who attend several courses may not be granted the expected recognition, but are compelled to accept jobs much below their qualifications resulting in job dissatisfaction^22^^,^^23^^,^^24^.

The literature includes many studies on stress, anxiety and depression among different populations. However, there are still gaps as concerns prevention and therapeutic strategies^25^, for which reason more studies are needed in this regard given the highly negative impact of these conditions on health and job performance. Furthermore, we call the attention to the relevance of studies which assess definite populations^26^ to determine the need for narrow-scoped, individualized measures according to the particular characteristics of each occupational group.

Since our sample exclusively comprised civil servants with job stability, comparisons to workers under the Labor Consolidation Law are relevant, more in particular within the current context of economic crisis and unemployment in Brazil. Under such circumstances, one may expect the latter group to exhibit higher levels of stress, anxiety or depression.

Finally, we suggest for future studies to perform comparisons between different groups of workers and higher education institutions, and to investigate financial issues which might behave as triggers of stress. In this way, discussions might be generalized and conclusions relative to different outcomes in different populations verified.

## CONCLUSION

The results indicate a high prevalence of anxiety and depression symptoms when the participants categorized as with possible and probable symptoms are considered together independently from their socioeconomic level. Rates were higher among the participants with higher educational level. The results further point to the need for interventions to improve the analyzed aspects among the target population and minimize the negative impact of symptoms on health.
